# Long-Term Cognitive Outcomes and Associated Quality of Life of Young Adults Who Experienced Liver Transplantation in Early Childhood

**DOI:** 10.3389/frtra.2022.919232

**Published:** 2022-07-07

**Authors:** Sue V. Beath, Zoe Taylor, Jo Wray, Charlotte Passingham, Carla Lloyd, Deirdre A. Kelly

**Affiliations:** ^1^The Liver Unit, Birmingham Women's and Children's Hospital, Birmingham, United Kingdom; ^2^Centre for Outcomes and Experience Research in Children's Health, Illness and Disability (ORCHID), Great Ormond Street Hospital for Children NHS Foundation Trust, London, United Kingdom

**Keywords:** cholestatic liver disease, psychosocial health, growth, adherence-compliance-persistance, transition

## Abstract

We evaluated long term outcomes in infants born between 1992 and 2002 with cholestatic liver disease (CLD) who underwent successful liver transplantation (LT). A total of 160 children with CLD were identified: 68 had developmental assessments before and after LT of whom 32 were excluded because they were followed up elsewhere; 16/36 consented to complete measures of IQ, anxiety, depression, health related quality of life (HRQoL), and a habits/employment survey. Illness severity and developmental attainment prior to LT were comparable with the 32 excluded and 20 patients who declined to take part. The IQ of young adults after LT (mean score = 91.13, range 75–108, SD 10.4) was not significantly improved compared to pre-LT scores (mean score = 85.7 range 50–111, SD 17), but was inversely correlated with stunting of growth and duration of disease before LT, highlighting the need for timely LT in CLD. HRQoL scores ranged from 22 to 99 (mean 64.5 SD 20.7), comparable to scores in other LT recipients. Five (31%) had mild-moderate depression; 5 (31%) had moderate-severe anxiety associated with reduced HRQoL (*P* = 0.01 and *P* = 0.06, respectively); and nine had problematic fatigue which correlated with reduced HRQoL (*r*^2^ = 0.4 *P* = 0.007). Reduced medication adherence was associated with fatigue (Spearman correlation *r*^2^ = 0.267; *P* = 0.09) and anxiety (Spearman correlation *r*^2^ = 0.597; *P* = 0.02). Raised body mass index was also associated with reduced and health-related quality of life scores PeLTQL© (*r*^2^ = 0.379 *P* = 0.011). Fifteen (94%) were undergoing education or were employed. The long-term neuro-cognitive and psychosocial outcomes of young adults transplanted as babies is encouraging, although anxiety/depression was more common than in the healthy population. Psychosocial questionnaires help identify those young adult LT recipients who may benefit from support.

## Introduction

Liver transplantation (LT) has been offered to babies since the 1980s, and the development of new surgical techniques, most notably the size reduction of a liver graft to the left lobe and the routine use of micro-surgical skills in vascular anastomoses (1), combined with improvements in nutritional and medical support has transformed the early and medium-term survival. From the outset, our unit measured the cognitive abilities of babies and older children at the time of admission for LT assessment, and family counseling was provided to facilitate psychosocial resilience (2). To plan appropriate therapy or intervention, neurocognitive and developmental skills were re-assessed at 5 and 10 years post-LT reviews.

The need for long-term immune suppression and regular monitoring means that even well-supported young adults may struggle to resume a normal educational and social life (3). This cohort of children transplanted between 1992 and 2002 have moved to adult health services, and this provides a unique opportunity to evaluate the impact of LT on these young people's neuro-cognitive abilities, their psychosocial health, and their current quality of life 16–23 years after transplantation.

The aim of this audit was to review recipients with liver transplant (with pre-transplant cholestatic diagnoses) at this key phase of life within the adult health sector to assess their long-term neurocognitive outcomes and quality of life using standard tools.

## Materials and Methods

### Permissions and Approvals

This audit was registered with the Clinical Audits and Registries Management Service at our institution in August 2017 (CARMS-00903).

### Audit Procedure

**Figure 1:**
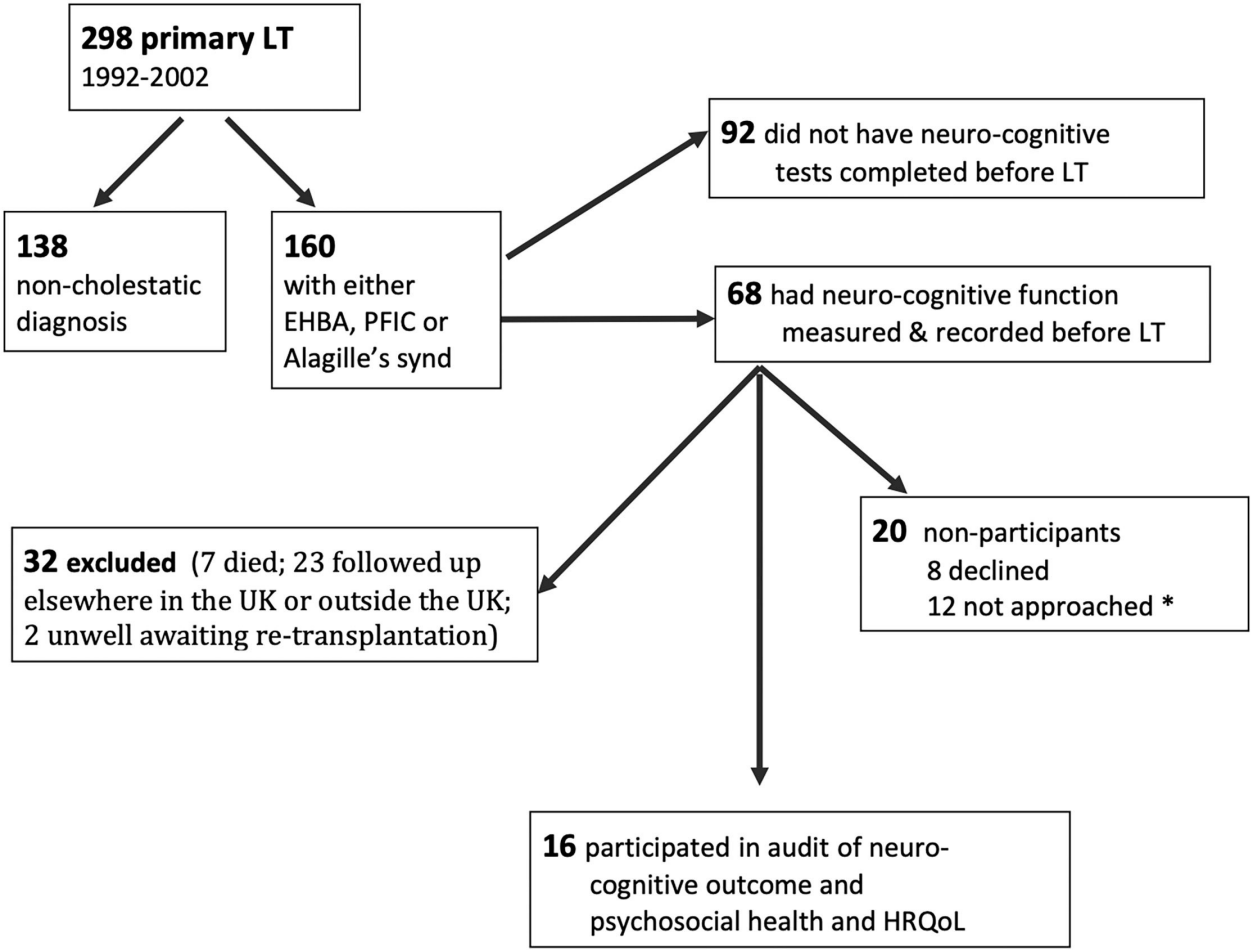
Flow sheet showing the recruitment process and availability of pre-transplant cognitive scores. *not approached because either appointments ran late/appointment was changed or psychologist was unwell on the day.

All children attending this regional Liver Unit between 1992 and 2002 with congenital cholestatic liver disease [biliary atresia (BA), alagilles, and progressive familial intrahepatic cholestasis (PFIC)] were identified from the liver transplant database. The eligibility criteria for inclusion in this audit were: completion of at least one pre-transplant neuro-cognitive assessment and being under follow-up in the liver clinic for young adults at either the pediatric site or the adult follow-up unit (both in the same city). Patients who were medically unstable at the time of the audit were excluded. The recruitment process is outlined in Figure 1. Out of 298 primary liver transplants carried out between 1992 and 2002, 138 were excluded (acute liver failure 23; autoimmune liver disease 14; liver tumor 14; acquired liver disease in 19, which includes drug drug-induced liver disease, graft vs. host disease, liver trauma; and metabolic liver disease 68, which includes 16 with cystic fibrosis and 19 with alpha-1-antitrypsin deficiency). Of the 160 identified with cholestatic liver disease from birth, 68 (42%) had pre-transplant cognitive tests recorded. Of this group, 7 had died and 23 were excluded because they were followed up at other centers (*n* = 21) or because they were awaiting re-transplantation (*n*=2). Thirty-six patients were eligible to participate.

### Participants and Process

Of the 36 eligible young adults, 24 were invited to complete the cognitive tests and other audit tools (see below). Twelve were not approached due to the following: the appointment changed, they arrived late and/or there was no time for testing, or the psychologist was unwell on the day. Eight declined to participate. Sixteen patientscompleted the cognitive testing and psychosocial questionnaires on the day of planned out-patient attendance. Data relating to demographic and clinical factors were obtained from the clinical database for participants. Data from the transition clinic included age at testing or review (for 20 non-participants), weight, height, details of immune suppression and liver function tests, and work or education status.

### Audit Tools

Pre-LT the Bayley scales were used to assess developmental function in all groups, except for eight children aged between 25 and 173 months, who had an age-appropriate Wechsler Intelligence Quotient test administered. Disease severity was measured using the parameters validated for the pediatric end-stage liver disease scores (PELD) (4). After LT, the Wechsler Abbreviated Scale of Intelligence (WASI-II) was used to measure cognitive function in the transition clinic. Anxiety and depression were measured with the Hospital Anxiety and Depression Scale (HADS) in 11, and the Beck Youth Inventories (BYI) in five aged 18 years or under. Fatigue was assessed using the Fatigue Severity Scale (5) and the quality of life was measured using the generic Euroqol EQ5D (6) and the condition-specific Pediatric Liver Transplant Quality of Life–[PeLTQL© (7)], which was minimally modified by our group (with permission from the author) to suit young adults over 17 years of age (more detail in Supplementary Table 1). In addition, the 16 participants completed a survey of habits and employment as part of a routine assessment for transition readiness, which included questions about adherence, use of alcohol and smoking, exercise, hospitalizations, education and employment, and relationship status.

### Statistics

Categorical data were compared using Fisher's exact test and continuous data were compared using independent *t*-tests or one-way analysis of variance to compare the pre-LT neuro-cognitive scores between participants and non-participants, and paired *t*-tests were used for comparisons of participants' scores over time. To facilitate comparisons between scores on the Bayley scales, the WPPSI, WISC, and WASI-II, raw scores were converted to a Z score (all scales have the same range and standard deviations). Correlations between clinical, demographic, and psychosocial measures were undertaken using Pearson or Spearman correlations or Fishers exact test as appropriate. All data were analyzed using the statistics package in Microsoft Excel version 16.54.

## Results

(a) Baseline data pre-LT (height, weight, liver function tests, and developmental), (see Table 1).

**Table 1:** Baseline demographic data pre-LT in a) 16 participants; b) 20 non-participants (declined to take part), and c) 32 excluded patients (7 died; 23 followed up elsewhere in the UK or outside the UK; 2 unwell awaiting re-transplantation).

	**Participants** **(*n* = 16)**	**Non-participants** **(*n* = 20)**	**Excluded** **(*n* = 32)**	**ANOVA *P*-value**
Gender (M:F) number percentage	6:10 38:62	11:9 55:45	17:15 53:47	0.29
Diagnoses number percentage with BA	BA, 15 Alagilles, 1 94%	BA, 18 PFIC, 2 90%	BA, 28 PFIC, 1 Alagilles, 3 87.5%	0.68
Median age pre-LT test months () = range; SEM	7 (3–73); 4.16	6.5 (3–173); 8.32	9.5 (4–172); 5.66	0.81
Cognitive function Pre-LT Mean total score (SD) Z score (SD)	85.7 (17) −0.95 (1.15)	88 (19) −0.75 (1.04)	84.5 (22) −1.03 (1.47)	0.80
Median age at LT months range = (); SEM	10 (6–73); 4.1	12 (5–175); 8.24	13.6 (5–173); 5.96	0.66
Median bilirubin μmol/L on day of LT () = range; SEM	166 (17–794); 49.3	201 (6–717); 48.4	162 (29–833); 57.9	0.97
Median PELD score on day of LT () = range; SEM	15 (−3 to 29); 2.5	9 (3–41); 2.53	13 (−6 to 42); 2.08	0.91
Median weight kg day of LT Median z score Z score range = (); SEM	9.9 −1.19 (−2.98 to 1.52); 0.36	8.6 −1.54 (−2.8 to −0.38); 0.16	9.45 kg −1.29 (−4.1 to −0.97); 0.23	0.44
Median height cm day of LT Median z score range = (); SEM	73.3 −1.48 (−3.7 to 1.12); 0.38	69.9 −1.88 (−5.2 to 1.6); 0.33	75.7 −1.46 (−4 to 2.2); 0.27	0.23
Median time in days spent in PICU post–LT range = (); SEM	2 (1–21); 1.19	3 (1–52); 2.87	2 (1–22); 1.17	0.10
Type of graft implanted (%)	Whole, 5 (31%) Split, 5 (31%) Reduced, 6 (38%)	Whole, 6 (30%) Split, 3 (15%) Reduced, 11 (55%)	Whole, 4 (13%) Split, 10 (31%) Reduced, 18 (56%)	0.21
Re-transplant required (%)	1/16 (6.25%)	3/20 (15%)	6^*^/32 (18.75%)	0.53

*BA, biliary atresia; PFIC, progressive familial intrahepatic cholestasis*.

* *3/6 re-transplants were followed by patient's death at 147 and 57 months later; the other three are alive*.

*Statistics including ANOVA, calculated using the statistics package in Microsoft Excel version 16.54*.

The demographic and pre-transplant cognitive data for the 16 participants, 20 non-participants, and 32 excluded patients are shown in Table 1. There were no significant differences between the three groups on any parameters.

(b) Follow-up data at the transition clinic, (see Table 2).

**Table 2:** Follow-up demographic data post-LT comparing participants and non-participants.

	**Participants (*n*=16)**	**Non-participants (*n*=20)**	***t*****-test,** ***P*** **-value**
Median age in years () = range; SEM	20.9 (17.7–28.5); 0.77	20.07 (17.1–34); 0.91	0.95
Median time in years since LT () = range; SEM	19.45 (16.1–23.2); 0.6	18.6 (15.9–24.6); 0.53	0.589
Median weight in kg Median BMI ()= range; SEM	67.8 24.5 (20–35); 1.19	61.3 22.6 (18–31); 0.95	0.12 0.077
Median height in cm Median height z score () = range; SEM	161 −0.93 (−4.5 to 2.3); 0.50	162 −1.76 (−4.1 to 0.66); 0.31	0.34
In work/education Not working or studying Missing data	15 (94) 1 (6)	7 (35) 2 (10) 11 (55)	
Fertility	1 child born	1 child born	
Median creatinine μmoles/L () = range; SEM	65 (44–114); 4.6	79 (47–150); 5.8	**0.03**
Median bilirubin μmoles/L () = range; SEM	11 (6–40); 2.4	12 (5–44); 2.2	0.58
Median Alkaline phosphatase IU/L () = range; SEM	83 (52–624); 37.2	126 (71–258); 15.9	0.61
Median alanine aminotransferase IU/L () = range; SEM	22 (8–117); 13.7	24 (15–91); 4.6	0.76
Immune suppression Calcineurin inhibitor Mofetil mycophenolate	14/20 4/20	16/20 7/20	0.55 0.53
Prednisolone none 5 mg or less	7 9^*^	8 12	0.83

* *One participant was on 10 mg prednisolone daily. Bold denotes p value reaching the significance level of less than 0.05 meaning that there is a less than 1 in 20 risk that that result occurred by chance*.

The demographic and clinical data including height and weight collected at post-LT follow-up did not differ between the participants and non-participants except for plasma creatinine, which was above normal for two subjects in the non-participant group (136 and 150 micromoles/L, respectively).

(c) Follow-up outcome data (FSIQ, PELTQL, and EQ-5D at transition clinic) and associations with age at LT, growth restriction at LT, and disease severity.

**Figure 2:**
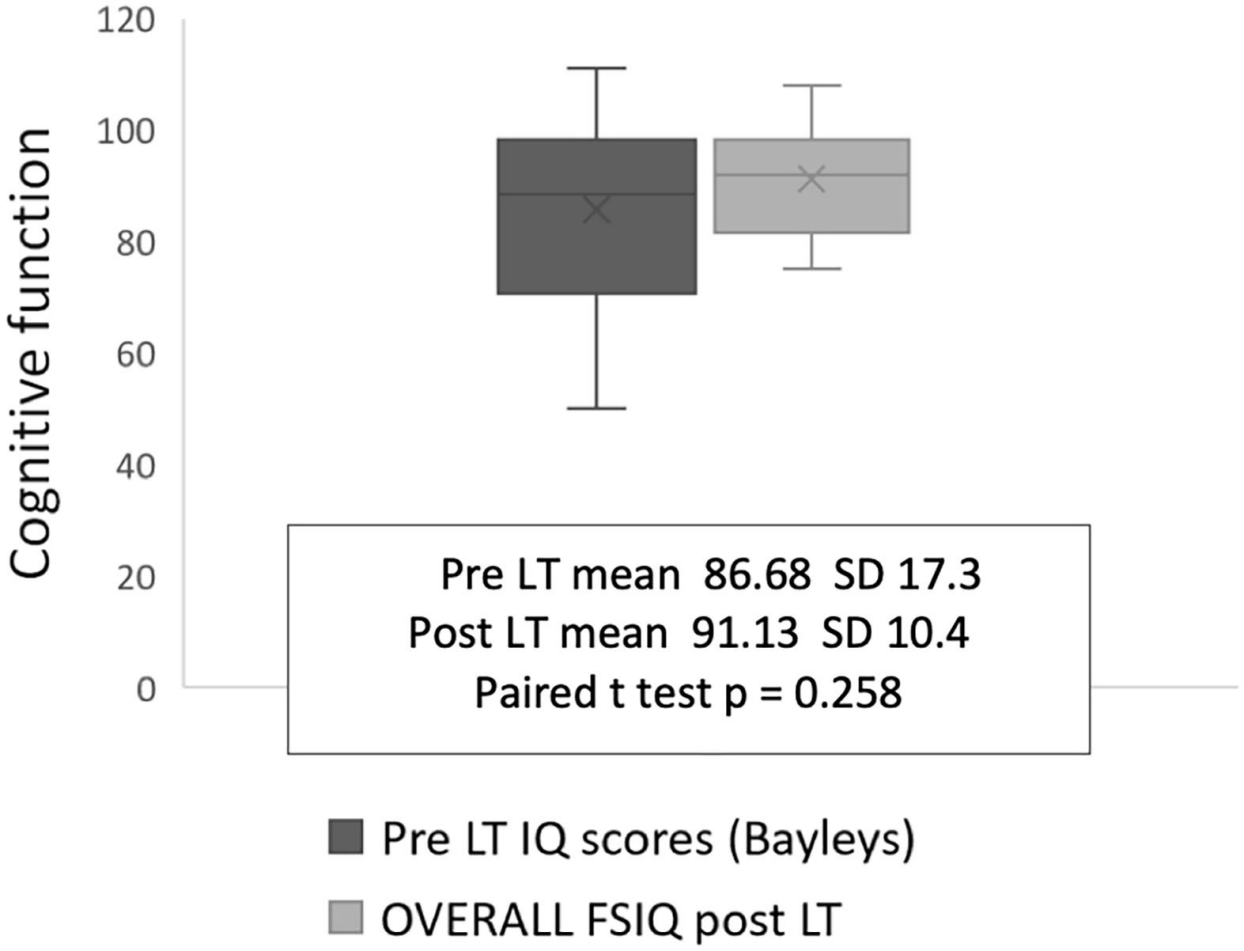
Cognitive scores pre and post-LT at transition.

There was no significant improvement or deterioration in cognitive performance for the participant group from pre-LT to post-LT, although some catch-up was present in four infants with low FSIQ scores between 75 and 80 (Figure 2). Although the Beck Youth inventory and HADS tools differ qualitatively, both categorize patients into one of four groups: normal, mild, moderate, or severe symptoms. Pooling the findings for the two measures showed that five individuals had depression (31%) and nine had anxiety (56%) at the time of testing in the transition clinic, (Table 3).

**Table 3:** Cognitive, psychosocial, fatigue, and HRQoL scores of young adult LT recipients at follow-up 16–23 years post-LT.

**Measure**	**Scores in transition clinic**	**Prevalence and range of values in normative populations**
Cognitive function ^*^ Mean ()= range; SD Z score; SD	91.13 (75–108); 10.4 −0.59; 0.69	Mean 100; normal range 80–120; SD 15
Depression scores BYI & HAD	11 normal; 3 mild; 2 moderate (31)**%**	11.3% of adolescents; 9.6% of young adults per year report major depressive episode (8)
Anxiety scores BYI & HAD	7 normal (44%); 4 mild; 3 moderate; 2 severe (56**%**)	14% of adults in the European Union per year experience anxiety (9)
Fatigue median raw score ()= range SEM	32.5 (15–50); 2.75	Healthy adults raw score = 21 or less Scores above 34 are considered indicative of problematic fatigue (4)
EuroQol 5D VAS Median/mean ()= range; SEM	81.6/81.5 (58–100); 3.3	Visual analog scale (VAS) = 0–100 https://www.york.ac.uk/che/pdf/DP172.pdf University of York UK reference for single people age under 25 years Mean score = 86.17 SD 13.7
PeLTQL© Median/mean ()= range; SEM	71.1/64.5 (22–95); 5.18	Scores <62.5 correlate with anxiety Scores <49.3 correlate with depression (7) Canadian LT recipients mean = 70.1

The * refers to, Part time work at same time as studying = 3.

The fatigue scale showed that four participants had scores comparable with those of a healthy reference group (raw score <27); six recorded scores between 27 and 36 (borderline fatigue) and six participants had global scores above 36, which is indicative of problematic fatigue. The mean value for the EQ5D visual analog score was 81.5 (SD 15), which is at the lower end for the population norm (10). The mean PeLTQL score was 64.5 (SD 20.7) with a range of 22–95 (LT reference range = 70.1 (SD12.4) (7). Seven participants had scores below 62.5, which is the threshold for being at a higher risk of anxiety in this group and five participants recorded scores below 50 (7). All seven participants with scores for higher risk of anxiety were given contact information for a liver disease patient welfare charity and four were referred to a Youth worker attached to the transition team. In addition, one was referred to a clinical psychologist and one was referred to mental health services in the adult sector.

**Figure 3:**
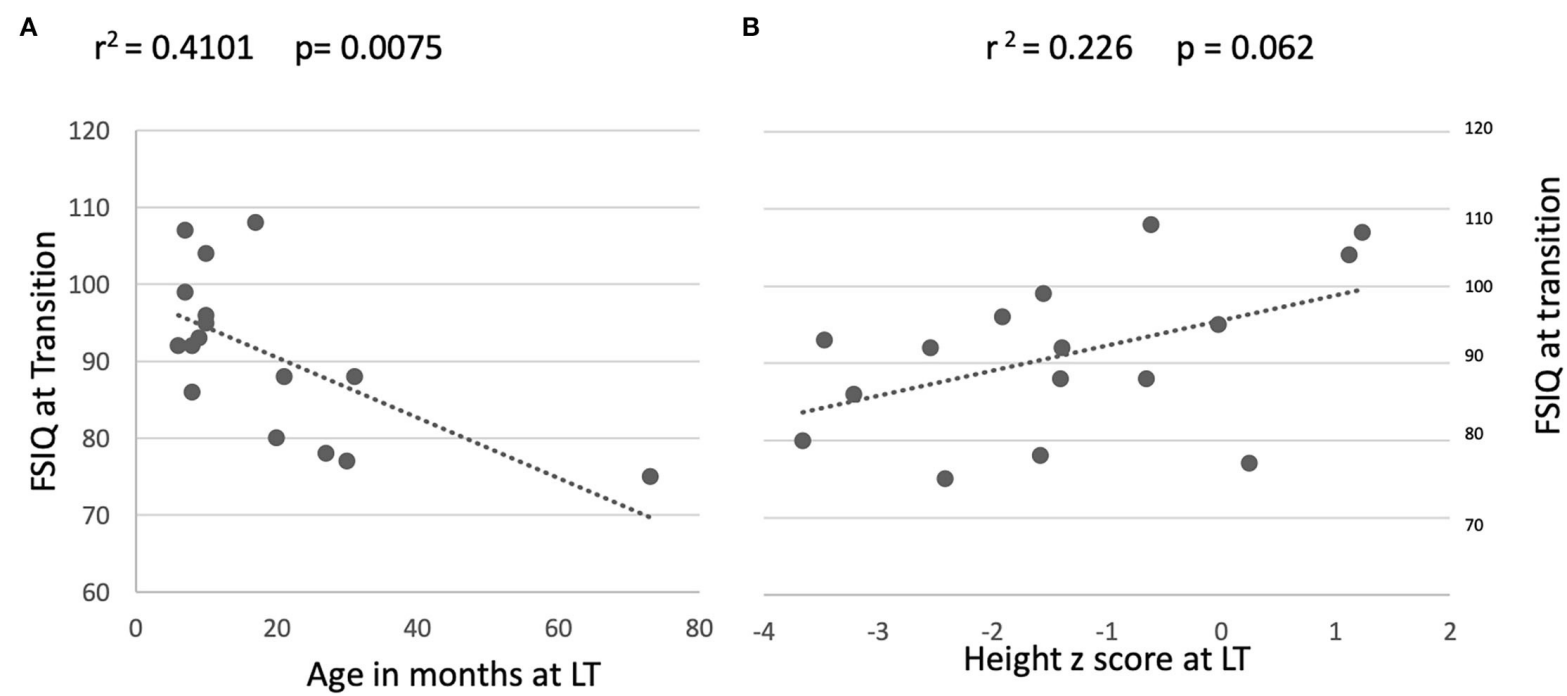
Relationship between long term FSIQ outcomes and age and height at transplant. **(A)** By age on day of transplant. **(B)** By height at transplant.

An association between older age at LT and lower FSIQ performance at post-LT follow-up was observed (*r* = 0.410 *P* = 0.008; Figure 3A). A trend toward restricted linear growth at the time of LT and lower FSIQ at follow-up in the transition clinic was also noted (*r* = 0.226 *P* = 0.062; Figure 3B). Other associations between disease severity markers (bilirubin, PELD, and days spent in intensive care post-operatively) and FSIQ, PeLTQL, and EQ-5D scores were not found (details available in Supplementary Table 2).

(d) Follow-up outcome data (FSIQ, PELTQL, and EQ-5D at transition clinic) and associations with mood and fatigue and body mass index (BMI).

**Figure 4:**
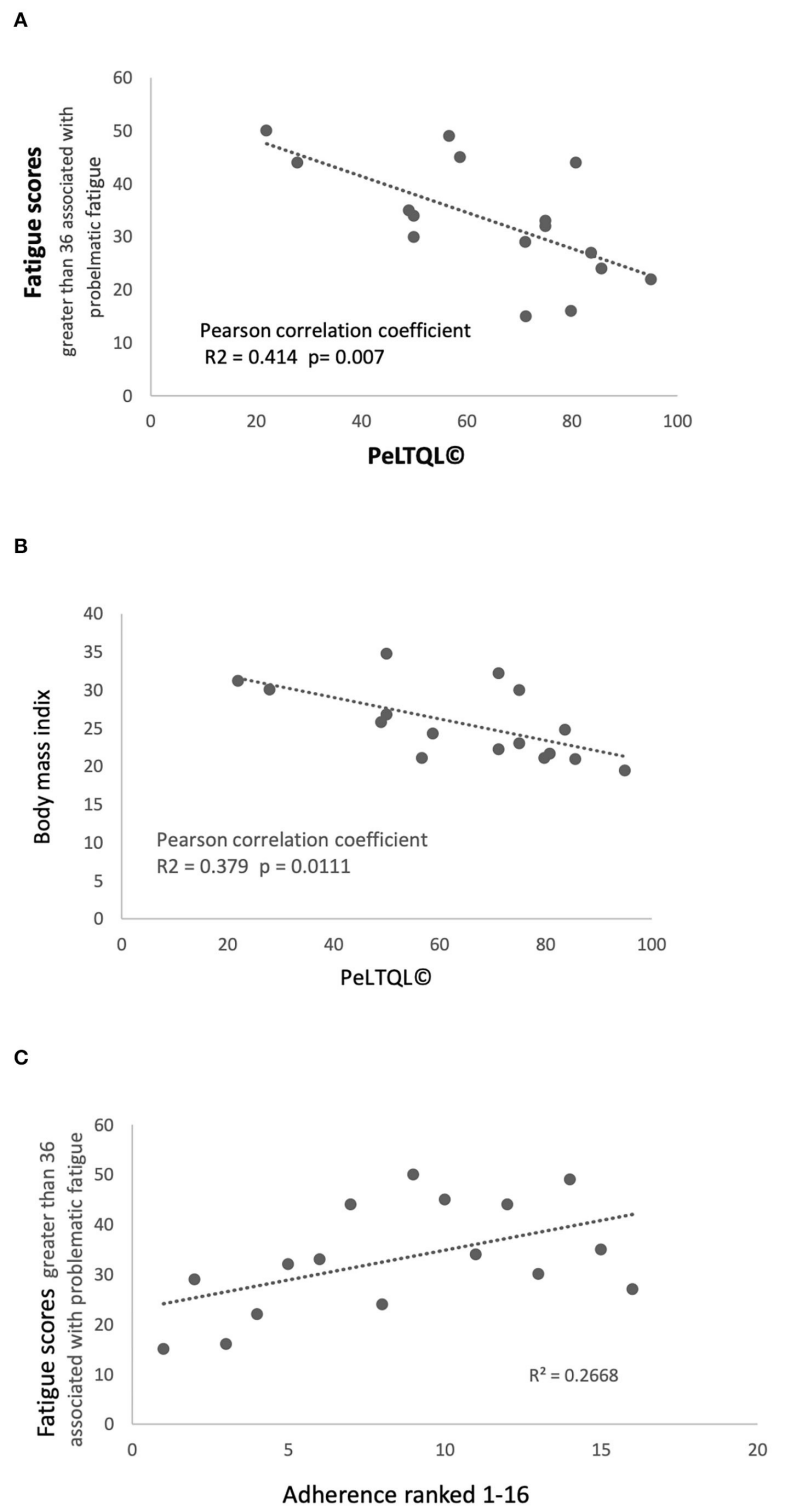
Fatigue scores, Health-related quality of life scores PeLTQL© and Adherence **(A)** Fatigue scores and PeLTQL©. **(B)** Raised body mass index and PeLTQL©. **(C)** Fatigue scores and Adherence.

There was no relationship between cognitive performance at follow-up and anxiety, depression, fatigue, or BMI, nor was there a significant association between cognitive performance and HRQOL (Supplementary Table 2). However, PeLTQL© showed a significant correlation with fatigue scores (PeLTQL© *r*^2^ = −0.414; *P* = 0.007; Figure 4A). Furthermore, lower PeLTQL© scores were associated with depression (Fisher's exact test 2-tailed *P* = 0.01) and anxiety (Fisher's exact test 2-tailed *P* = 0.06). Raised BMI was associated with lower PeLTQL© scores *r* = −0.339 *P* = 0.0111; Figure 4B).

(e) Survey of habits and employment,

Two participants declared that they never forgot their medications and the other 14 admitted forgetting to take immune suppression (IS) in varying degrees (Table 4). Eight had been hospitalized in the previous 5 years, although only three patients with liver-related problems. Smoking, alcohol consumption, relationship status, education, and employment are shown in Table 4. An association between forgetting to take IS medication and increased anxiety (*r*^2^ = 0.597; *P* = 0.02) and fatigue was found (*r*^2^ = 0.267; *P* = 0.09; Figure 4C). Lower scores on the PeLTQL© (<62.5) were associated with reduced adherence (*P* = 0.011 by Fisher's exact test). There were no associations between forgetting IS medication and abnormal liver function, raised creatinine, or a history of hospitalizations (Supplementary Table 3).

**Table 4:** Life-style survey results at follow-up.

	**Number (%)**	**Details**	
Forgot to take medications	No = 2 (13%) Yes = 14 (87%)	Never Rarely, 1–4 times/year Occasionally, 2–4 times/month Often, 2–4 times/week	2 4 5 5
Smoke	No = 14 (87%) Yes = 2 (13%)	3 cigarettes/day; 8 cigarettes/day, respectively	
Alcohol	None = 11 (69%) Yes = 5 (31%)	Range of consumption reported: 2–12 units/week	
Exercise	No = 1 (7%) Yes = 15 (93%)	None Not specified 1–2 times per month 1–2 times per week 3 times per week 5 times per week	1 1 2 8 3 1
Education and	No = 1 (7%) Yes = 8 (50%)^*^	Neither in work or education Attending college Attending university	1 5 3
Employment	Yes = 11 (69%)^*^	Office work Retail & leisure Manual ^*^Part time work at same time as studying = 3	4 4 3
Relationship	No = 8 (50%) Yes = 8 (50%)	8 single; 8 in relationship including one with a child	
Contraceptive measures	No = 7 (44%) Yes = 9 (56%)	Coil Oral contraceptive pill Implant Condom Other none	4 2 1 1 1 7

The * refers to, Part time work at same time as studying = 3.

## Discussion

This is the first report in which long-term medical outcomes have been evaluated in association with the evaluation of formal cognitive testing, psychosocial, quality of life, and lifestyle outcomes in a group of young people transplanted in the early years of liver transplantation and followed up in a single center. We choose to focus on those children born with cholestatic disorders because the degree of malnutrition caused by the associated fat malabsorption is more extreme than in children with acute liver failure or other disorders acquired later in childhood such as autoimmune hepatitis, liver tumors, etc. Additionally, we hypothesized that cognitive outcomes might be impacted by fat malabsorption experienced before LT through the mechanism of calorie restriction and other key nutrients, such as fat-soluble vitamins and omega 3 fatty acids, which are required for incorporation in neural membranes (10, 11).

Obtaining longitudinal data over a time scale of 16–25 years was challenging (12). Despite our intention to record the cognitive abilities of all babies and children undergoing LT for chronic cholestatic liver disease, the constraints of time (some children needed urgent placement on the transplant waiting list), ongoing medical care, space, and reductions in staffing meant that pre-transplant neuro-cognitive testing was performed in only half the eligible children. Most of the survivors eligible for this audit have moved to different health service providers and could not be contacted. Despite considerable effort and goodwill from the young people and our adult colleagues, we were only able to complete the audit cycle in 16/36 suitable participants. However, the 16 tested participants are representative of the group, as a whole, since their diagnoses and transplant experiences are comparable with those patients who did not participate. In an additional effort to ascertain the comparability of the tested group with those we were unable to assess, we compared the pre-transplant disease severity and pre-LT neuro-cognitive performance in those who declined to be tested/missed appointments and those who were no longer followed up locally with the participant group, and found all 3 groups to be comparable (Table 1).

Even though this data set is small, it is unique as few studies report such a long-term paired neurocognitive outcome. We found an association between older age at LT (more than 2 years) and suppressed linear growth pre-LT and lower FSIQ performance at follow-up 16–23 years later, which supports the view that the long duration of symptomatic cholestatic liver disease before LT (more than 2 years) affects neurocognitive function in adult life, Figure 2, as we described previously [9]. This hypothesis is reinforced by the findings of Leung et al. who, in studying a large cohort of children who still have their native livers, found that malnutrition and liver disease severity were significantly associated with FSIQ (13). The impact of liver disease severity just prior to LT in our patients was less obvious since bilirubin and albumin concentration, PELD, and post-operative days spent in PICU did not correlate with long-term FSIQ or the quality of life at the follow-up clinic. Whilst we might have expected to see improvements in FSIQ once the liver disease was treated by transplantation, in fact, we found that only four participants appeared to benefit, and, for the group as a whole there was no significant change in FSIQ.

The finding that HRQoL is relatively good is an encouraging outcome, despite the acknowledged physical and psychological impacts of transplantation on the young person (3). However, our patients reported higher levels of depression and anxiety after LT than in previous reports (14), which identified 9.8% of 51 young adults (post-LT) had depression; 17.7% had anxiety, compared to 31% with depression and 56% with anxiety in our patients. It is possible that these differences may be related to using different measurement tools, but there is a general rise in depression and anxiety in the adult populations of Europe and North America (8, 9) and a rising prevalence of depression globally (15). Although the Adolescent Support team for patients being prepared to transfer over to adult services in our unit is proactive in asking screening questions related to transition readiness, including a question about telling the doctor or nurse how you are feeling (https://www.rheumatology.org/Portals/0/Files/Transition-Readiness-Assessment-Questionnaire.pdf), the results in this study suggest that additional specific questions about symptoms of anxiety/depression should be standard. We believe such screening is important not just for quality of life, but also because mental health problems were reported to be risk factors for non-adherence in at least two studies (16, 17), and this is a concern for the long-term health of the patient and his/her liver graft.

Our study found that irregularities in taking tacrolimus were disclosed by 87.5% participants (at least once or twice per month in 10/16) and that the greater degree of non-adherence was associated with greater anxiety, which was also linked to lower quality of life scores *via* several factors including raised body mass index and fatigue. This degree of non-adherence is high compared to a younger age group aged 0–17 year, where irregularities in taking tacrolimus were reported in 34% (95% confidence interval = 30–39%) of children aged 0–17 years (18). It confirms the observation that transitioning to adult services coincides with a tendency to forget to take medications. We also found that higher fatigue scores were associated with an increased frequency of forgetting to take IS, which may have contributed to our participants' sense of anxiety. The use of an HRQoL tool, which detects fatigue and anxiety, could be an effective way of identifying patients who need extra help at the time of transition to adult services. Other factors which have been reported to influence HRQoL in large cohorts, such as the history of a complicated transplant course and the duration of time spent in intensive care were not identified (19), possibly because more than 16 years (median 20 years) have elapsed since the transplant episode in this study.

The main strength of the study is the consistent follow-up of this patient cohort before and after LT in one center. However, we acknowledge the following concerns: the Bayley scale is one of the best-validated tools for measuring cognitive abilities in infants, but the measures do not correspond well with the Weschler intelligence tests used in older children and young adults, which makes it difficult to evaluate longitudinal development and IQ. Secondly, to measure anxiety and depression across an age range 16–26 years, we had to pool data from two separate age-appropriate tests: the Beck Youth Inventory and the Hospital Anxiety and Depression scale; thirdly, there was only one condition-specific quality of life tool, the PeLTQL, which was developed for 8–17 years old at least 1 year post-transplant (7). We had to modify it for use in our young adult cohort by changing the wording of three of the items, but according to the PeLTQL Manual and Interpretation Guide this adjustment, if limited to just three questions, it does not affect the validity of the tool.

In conclusion, we found in this audit of long-term outcomes of LT performed in early childhood that neurocognitive function was satisfactory and catch-up function was achieved in those whose score was >2 SD below the mean before LT. The prevalence of anxiety and depression was higher than reported in other LT cohorts (using a different tool), and HRQoL was a little lower than reported previously in adolescent survivors (7). The lifestyle survey demonstrated that 16–23 years post-transplant, most survivors were engaged in either education or work, half were in a relationship with a partner, and three-quarters were exercising once or more per week. We recommend regular exercise and/or physical rehabilitation not just for its benefits to quality of life, but also for improved mental health (20) and, potentially, improved adherence in LT recipients. We support recommendations for multi-professional teams to deploy condition-specific screening tools, such as the PeLTQL, when preparing a young person for transition to adult services, together with psychosocial screening measures, particularly as these psychological states may affect adherence and wellbeing (16). Early recognition of mental health disorders combined with a multi-professional approach with input from psychologists, nurse specialists, physiotherapists, and physicians with expertise in the management of young adults with chronic disorders is essential in helping more survivors of LT in early childhood attain healthy adult autonomy with a good quality of life.

## Data Availability Statement

The original contributions presented in the study are included in the article/Supplementary Material, further inquiries can be directed to the corresponding author/s.

## Ethics Statement

Ethical review and approval was not required for the study on human participants in accordance with the local legislation and institutional requirements. Written informed consent from the participants' legal guardian/next of kin was not required to participate in this study in accordance with the national legislation and the institutional requirements.

## Author Contributions

SB wrote the first draft, created the tables and figures, and edited subsequent drafts. DK had the original idea to systematically record cognitive and other parameters pre transplant with the aim of reporting long term outcomes, and, in addition to designing the audit, provided intellectual input, and critical reviews of the manuscript in all its stages and final conclusions. CL contributed to identification of participants *via* the transplant registry and to data collection and retrieval of demographic data before and after transplant and liaised with staff in the transition clinics. CP contributed to data collection and retrieval of demographic data especially height and weight and clinical biochemistry before and after transplant. JW contributed to the design of the audit, corrected and amended later drafts of the manuscript especially providing intellectual input to interpretation of the data and conclusions. ZT assessed the participants, collected and collated the pre and post-transplant cognitive data, administered the questionnaires relating to quality of life, anxiety and depression, and life style survey and created the spreadsheet. All authors contributed to the article and approved the submitted version.

## Funding

The author's salaries were paid by the National Health Service and the work was done as a departmental audit accommodated within each person's job plan. Permissions and Approvals were given by the audit department at Birmingham Children's Hospital, Steelhouse Lane, Birmingham, B4 6NH, United Kingdom. This audit was registered with the Clinical Audits and Registries Management Service August 2017 (CARMS-00903). £3000 funding was made available by the SPLIT charity https://splituk.org (charity no 1111945) to cover: the cost of licenses to use questionnaires; any added travel expenses incurred by patients whose out-patient department appointment over ran because of participation; fees for retrieval of archived notes from external storage facility.

## Conflict of Interest

The authors declare that the research was conducted in the absence of any commercial or financial relationships that could be construed as a potential conflict of interest.

## Publisher's Note

All claims expressed in this article are solely those of the authors and do not necessarily represent those of their affiliated organizations, or those of the publisher, the editors and the reviewers. Any product that may be evaluated in this article, or claim that may be made by its manufacturer, is not guaranteed or endorsed by the publisher.

## Abbreviations

BA: biliary atresia
BMI: body mass index
BYI: beck youth inventories
CARMS: Clinical Audits and Registries Management Service
CLD: cholestatic liver disease
EQ5D: Euroqol 5 domain tool
FSIQ: full scale intelligence quotient
HADS: hospital anxiety and depression scale
HRQoL: health related quality of life
IQ: intelligence quotient
LT: liver transplantation
PELD: pediatric endstage liver disease
PeLTQL: pediatric liver transplant quality of life
PFIC: progressive familial intrahepatic cholestasis
WASI-II: wechsler abbreviated scale of intelligence
WISC: wechsler intelligence scale for children
WPPSI: wechsler pre-school and primary scale of intelligence.
