# Supplementary material for: Long-Term Cognitive Outcomes and Associated Quality of Life of Young Adults Who Experienced Liver Transplantation in Early Childhood
Source: Front Transplant. 2022 Jul 7;1:919232. doi: 10.3389/frtra.2022.919232 (PMC11235375; doi:10.3389/frtra.2022.919232)
Supplement: Supplementary file 1 [file Table_1.pdf]

Supplementary Table 1 Details about tests administered to the audit groups

Test	What it measures and in which participant group	How long the test takes to administer / other factors affecting utility
Bayley scales of infant and toddler development	Standard test of development for infants and toddlers aged 1-42 months; enables identification of strengths and competencies as well as weaknesses;	Requires face to face test conditions, and qualified psychologist to administer (training required). Takes 45-60 minutes. License to use required; kit available to purchase from Pearson
Wechsler scales	Widely used measure of intelligence with age-appropriate versions from preschool through to adult; the Wechsler Abbreviated Scale of Intelligence (WASI) is a short form estimate of intellectual functioning suitable for children from the age of 6 years through to adults of 90 years; the WASI comprises two or four subtests	Administered by registered user face to face; WASI 4 subtest version (30 minutes to administer) comprises vocabulary, block design, similarities and matrix reasoning and a full scale IQ (FSIQ) is computed. license to use required and can be purchased from Pearson
Beck Youth Inventory (BYI)	Five inventories to evaluate emotional and social functioning in children and young people aged 7-18 years; inventories each contain 20 statements about thoughts, feelings and behaviours and the respondent rates how frequently each statement has been true for them; inventories cover depression, anxiety, anger, self-concept and disruptive behavior; anxiety and depression inventories used in the current audit; scores are classified as severe, moderate, mild or no problems	Takes 5-10 minutes to complete each inventory; available to purchase from Pearson
Hospital Anxiety and Depression Scale (HADS)	A 14 item measure of anxiety and depression (7 items each) developed for people with physical health problems; each item is scored from 0-3, with a maximum score of 21 for each the two subscales; scores are classified as severe, moderate, mild or no problems	Completion takes 5-10 minutes; available to purchase from GL Assessment
Fatigue severity scale (FSS)	A 9 item scale measuring the severity of fatigue and the impact of it on activities and lifestyle. Responses are on a 7 point scale indicating level of agreement; can be used with children and young people of 8 years and older	Takes 5 minutes<br>No training required; can be done by post or over the telephone. Free to use.
EQ-5D	A generic standardized measure comprising one question in each of 5 domains (mobility, self-care, usual activities, pain/discomfort and anxiety/depression) assessed on a 5 point scale and a visual analogue scale for respondents to evaluate overall health status; can be used in young people 16 years old & above	Takes 5 minutes<br>No training required; can be done via post or over the telephone. Free to use in audit of less than 100 participants
PeLTQL©	Disease-specific questionnaire for children and young people aged 8-17 years who have undergone LT at least 12 months previously; 26 items in 3 domains: Future health (9 questions); coping and adjustment (8 questions) Social-Emotional (9 questions); 5-point Likert scale used and responses reverse scored and scaled to provide possible final scores with a range of 0-100, with higher scores indicating better HRQoL	Takes 5-10 minutes to complete; easy to administer and complete; some training required for scoring and administration. Copyright protected – permissions need to be obtained from PeLTQL.info@sickkids.ca Minimal revisions were made to the wording of 3 questions to make the measure appropriate for young people over the age of 17 years, as follows: *Question 5 - "How do you feel about changing to a health care team that takes care of adults when you get older?" was changed to "How did you feel about changing to a health care team that takes care of adults?"; Question 10 - "Do you feel that your parents are too protective?" was changed to "Do you feel your family are too protective?"; Question 18 - "Do you worry that you miss more school than other students who have not had a liver transplant" was changed to "Do you worry that you have missed more school or college than other people who have not had a liver transplant?"*
