# Supplementary material for: Long-Term Cognitive Outcomes and Associated Quality of Life of Young Adults Who Experienced Liver Transplantation in Early Childhood
Source: Front Transplant. 2022 Jul 7;1:919232. doi: 10.3389/frtra.2022.919232 (PMC11235375; doi:10.3389/frtra.2022.919232)
Supplement: Supplementary file 2 [file Table_2.pdf]

Supplementary table 2 Clinical variables on day of LT (items 1-6) and psychosocial measures at follow-up (items 7-11) correlated with cognitive and quality of life outcomes at post-LT follow-up

		FSIQ	PeLTQL©
1.	Bilirubin day of LTx	R^2= 0.009<br>p = 0.727	R^2 =0.203<br>p = 0.08
2.	PELD on day of LT	R^2 = 0.035<br>p = 0.487	R^2 = 0.014<br>p = 0.661
3.	Post – op days in PICU	R^2 = 0.004<br>p = 0.487	R^2 = 0.003<br>p = 0.834
4.	Age at LT	**R^2 = 0.414<br>p = 0.008**	R^2 = 0.028<br>p = 0.834
5.	Weight z score at LT	R^2 = 0.048<br>p = 0.413	R^2 = 0.011<br>p = 0.700
6.	Height z score at LT	**R^2 = 0.226<br>p = 0.063**	R^2 = 0.045<br>p = 0.429
7.	Weight z score at transition	R^2 = 0.06<br>p = 0.347	**R^2 = 0.385<br>p = 0.01**
8.	Height z score at transition	**R^2 = 0.267<br>p = 0.041**	R^2 = 0.093<br>p = 0.246
9.	Body mass index at transition	R^2 = 0.0005<br>p = 0.937	**R^2 = 0.379<br>p = 0.011**
10.	Depression BYI & HADS	p = 0.596[a]	**p = 0.001[b]**
11.	Anxiety BYI & HADS	p = 1.00[a]	**p = 0.059[c]**
12.	Adherence (lower adherence associated with lower PeLTQL©)	p = 0.302	**p = 0.093[b]<br>p = 0.011[c]**
13.	Fatigue	R^2= 0.02<br>p = 0.59	**R^2 = 0.414<br>p = 0.007**
14.	EQ-5D visual analogue scale	R^2 = 0.049<br>p = 0.411	R^2 = 0.146<br>p = 0.144
15.	PeLTQL©	R^2 = 0.021<br>p = 0.59	

[a]Fishers exact test using mean FSIQ 91 as a threshold
[b]Fishers exact test using PeLTQL score of 49.3 as a threshold (as a threshold for depression)
[c]Fishers exact test using PeLTQL score of 62.5 as a threshold (for anxiety)
Other correlations calculated using Pearson correlation coefficient
