# Supplementary material for: Long-Term Cognitive Outcomes and Associated Quality of Life of Young Adults Who Experienced Liver Transplantation in Early Childhood
Source: Front Transplant. 2022 Jul 7;1:919232. doi: 10.3389/frtra.2022.919232 (PMC11235375; doi:10.3389/frtra.2022.919232)
Supplement: Supplementary file 3 [file Table_3.pdf]

Supplementary table 3

Correlations between self reported adherence and psychosocial health and health related quality of life

	Adherence
Fatigue [a]	**R^2 = 0.267**<br>**p = 0.03**
EQ-5D visual analogue scale [a]	R^2 = 0.197<br>p = 0.084
PeLTQL© [a]	R^2 = 0.10<br>p = 0.234
Body mass index [a]	R^2 = 0.02<br>p = 0.589
Abnormal liver function tests [a]	R^2 = 0.163<br>p = 0.135
Creatinine [a]	R^2 = 0.034<br>p =0.49
Hospitalisations [b]	p = 0.281
Exercise [c]	p = 1.000
Depression<br>as scored in BYI and HADS [d]	R^2 = 0.409<br>p = 0.2
Anxiety<br>as scored in BYI and HADS [d]	**R^2 = 0.597**<br>**p = 0.02**

[a] Pearson regression analysis

[b] Fishers exact test using none versus hospitalization episode; and never or rarely forget medicines versus occasional or often as thresholds in 2x2 contingency table

[c] Fishers exact test using once a week or less versus more than once a week of exercise; and never or rarely forget medicines versus occasional or often as thresholds in 2x2 contingency table

[d] Spearman rank correlation
