# Supplementary material for: Long-Term Cognitive Outcomes and Associated Quality of Life of Young Adults Who Experienced Liver Transplantation in Early Childhood
Source: Front Transplant. 2022 Jul 7;1:919232. doi: 10.3389/frtra.2022.919232 (PMC11235375; doi:10.3389/frtra.2022.919232)
Supplement: Supplementary file 4 [file Presentation_1.PPTX]

## Slide 1
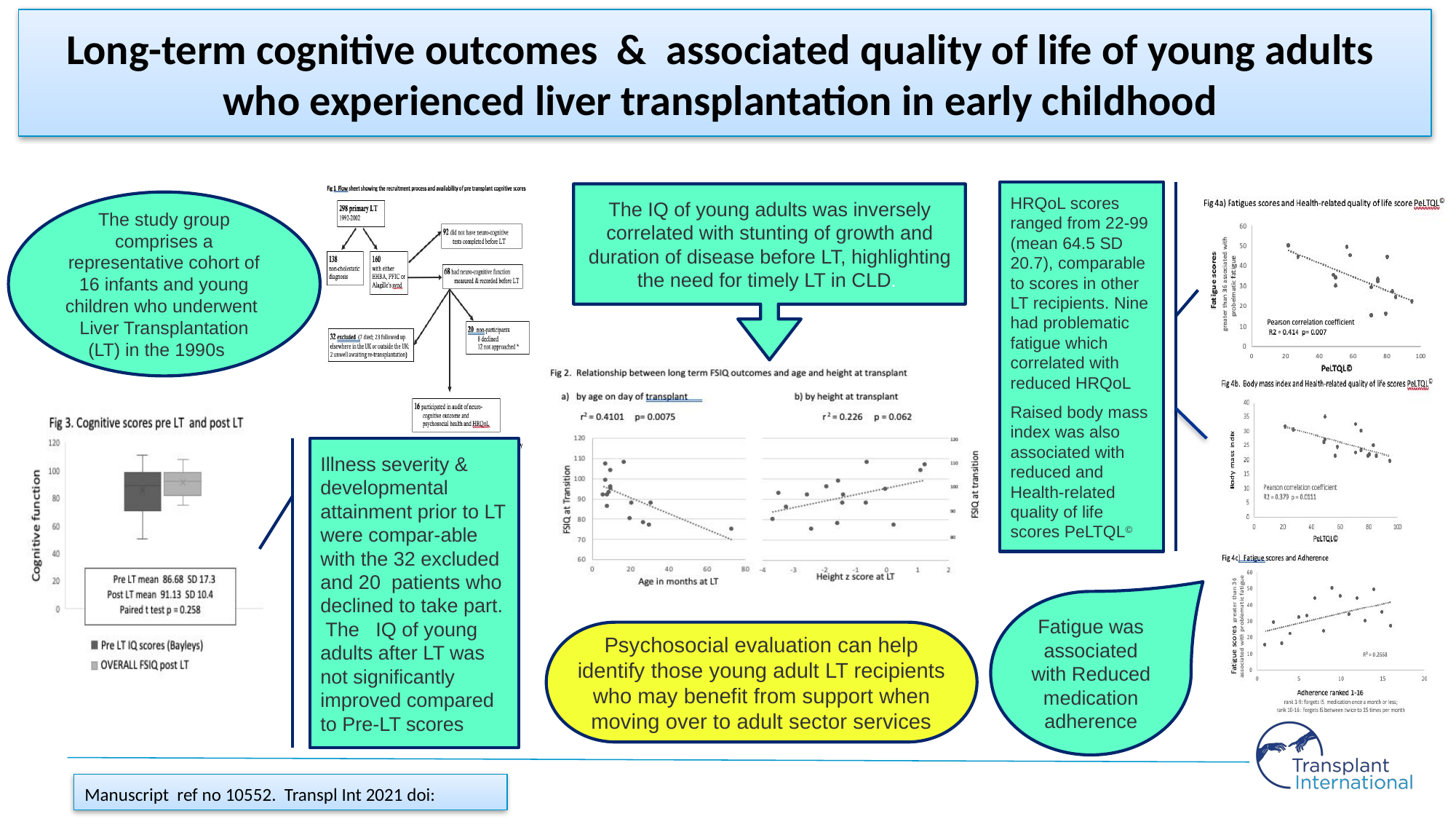

# Long-term cognitive outcomes & associated quality of life of young adults who experienced liver transplantation in early childhood
HRQoL scores ranged from 22-99 (mean 64.5 SD 20.7), comparable to scores in other LT recipients. Nine had problematic fatigue which correlated with reduced HRQoL
Raised body mass index was also associated with reduced and Health-related quality of life scores PeLTQL©
The IQ of young adults was inversely correlated with stunting of growth and duration of disease before LT, highlighting the need for timely LT in CLD.
The study group comprises a representative cohort of 16 infants and young children who underwent Liver Transplantation (LT) in the 1990s
Illness severity & developmental attainment prior to LT were compar-able with the 32 excluded and 20 patients who declined to take part. The   IQ of young adults after LT was not significantly improved compared to Pre-LT scores
Fatigue was associated with Reduced medication adherence
Psychosocial evaluation can help identify those young adult LT recipients who may benefit from support when moving over to adult sector services
Manuscript ref no 10552. Transpl Int 2021 doi:
